# Idiopathic pyometra and tubo-ovarian abscess in a postmenopausal patient treated conservatively

**DOI:** 10.3205/000311

**Published:** 2022-06-14

**Authors:** Maria Ntioudi, Katerina Vasiliadou, Parthena Charalampidou-Keremidou

**Affiliations:** 1 Gynecology – Obstetrics Department, General Hospital of Pella, Hospital Unit of Edessa, Greece

**Keywords:** pyometra, tubo-ovarian abscess, postmenopausal

## Abstract

**Background:**

Pyometra is a rare gynecological condition and is characterized by pus accumulation in the uterine cavity. It occurs more frequently in postmenopausal women than tubo-ovarian abscesses, which constitute a more common gynecological complication among premenopausal women.

**Objective:**

A 72-year-old woman was admitted to our emergency department with lower abdominal pain, diarrhea and fever for the last three days. The laboratory results were indicative to sepsis. The clinical examination revealed sensitivity by palpation of the lower abdomen without any signs of acute abdomen. The gynecological assessment showed pus outflow through the cervix and a pus culture was done. The ultrasound examination found an enlarged uterus, full of hypoechoic fluid, unclear borders between endometrium-myometrium, a mixed echogenicity adnexal mass and no free fluid in the pouch of Douglas. A computed tomography (CT) of the abdomen showed the presence of pyometra and a tubo-ovarian abscess of the right adnexa.

**Method:**

The patient was treated with intravenous antibiotic therapy. When the patient was hemodynamically stable and afebrile, she underwent ultrasound-guided dilatation and curettage of the cervical canal and the endometrium in order to exclude an underlying malignancy, under general anesthesia.

**Results:**

The patient responded promptly to the intravenous antibiotic therapy which was adapted to the pus culture result. The laboratory results withdrew to normal values and the patient was discharged after fifteen days of hospitalization in an afebrile and hemodynamically stable condition.

**Conclusion:**

Pyometra and tubo-ovarian abscess in postmenopausal women could be a lethal complication of pelvic inflammatory disease. The key in treatment is the dilatation of the cervix and drainage of the pyometra. The administration of intravenous antibiotics and drainage through the cervix could be a suitable method of treatment for pyometra in older patients or those with poor performance status if only the histological examination is negative for malignancy.

## Zusammenfassung

**Einleitung:**

Pyometra ist eine seltene gynäkologische Erkrankung und wird als eine Eitersammlung im Uterus beschrieben. Sie wird in der Regel bei postmenopausalen Frauen diagnostiziert, im Gegensatz zum Tuboovarialabszess, der eine häufige Erkrankung bei prämenopausalen Frauen darstellt.

**Anamnese:**

Eine 72-jährige Frau stellte sich in der Notaufnahme mit unteren abdominellen Schmerzen, Diarrhöen und Fieber seit drei Tagen vorg. Die Laborergebnisse wiesen auf eine Sepsis hin. Die klinische Untersuchung zeigte einen eitrigen vaginalen Ausfluss, und ein vaginaler Abstrich wurde entnommen. Die Ultraschalluntersuchung zeigte einen vergrößerten Uterus, der mit echoarmer Flüssigkeit gefüllt war, das unscharf begrenzte Endometrium und einen inhomogenen Ovarialtumor ohne freie Flüssigkeit im Douglas. Die Computertomographie (CT) des Abdomens zeigte eine Pyometra und einen Tuboovarialabszess des rechten Ovars.

**Methode:**

Die Patientin wurde mit einer intravenösen, antibiotischen Therapie behandelt. Nach hämodynamischer Stabilisierung erfolgte die sonographisch gesteuerte Dilatation und Abrasio der Zervix und des Endometriums in Allgemeinanästhesie, um eine Malignität auszuschließen.

**Ergebnisse:**

Die Patientin hat rasch auf die intravenöse antibiotische Therapie angesprochen, die im Verlauf antibiogrammgerecht umgesetzt wurde. Die Laborwerte haben sich normalisiert und die Patientin konnte nach einem stationären Aufenthalt von 15 Tagen kardiopulmonal stabil und afebril entlassen werden.

**Diskussion:**

Pyometra und Tuboovarialabszess bei postmenopausalen Frauen kann eine letale Komplikation der entzündlichen Erkrankung des Beckens (PID) sein. Der wichtigste Punkt der Therapie ist die Dilatation der Zervix und die Entlastung des Eiters. Die Verabreichung einer antibiotischen Therapie und die Drainage kann die entsprechende Therapie von Pyometra bei älteren Patientinnen oder bei jenen mit eingeschränktem Gesundheitszustand sein, wenn die Histopathologie keinen Nachweis einer Malignität ergibt.

## Introduction

Pyometra is a rare gynecological condition that affects 0.01–0.05% of women, mainly those of postmenopausal age [1]. It is characterized by the accumulation of pus in the uterine cavity. Several causes could lead to pyometra, such as a cervical or an endometrial malignancy, prior pelvic radiation and, in addition, benign conditions, specifically leiomyomas, endometrial polyps, congenital anomalies, occlusion of the cervix after surgery and retention of an IUD (intrauterine device) [2], or it could be idiopathic [3]. Pyometra manifests with lower abdominal pain, postmenopausal bleeding and vaginal discharge [4]. It is a consequence of a blocked cervical canal [5]. As compared with pyometra, tubo-ovarian abscesses are less common in the postmenopausal population, and are estimated to amount to 6 to 18% of the total cases reported [6]. The risk factors diverge from those of premenopausal patients and include recent surgical procedures and more particularly endometrial sampling [7], comorbidities such as diabetes mellitus (DM), HIV [8] and the retention of an IUD [9].

## Case description

**Figure 1:**
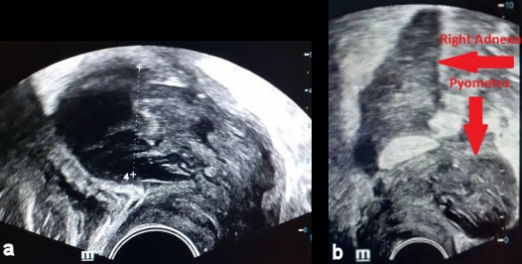
Transvaginal ultrasound (TVUS) a) of the uterus in the sagittal plane, b) of the right adnexa in the sagittal plane

**Figure 2:**
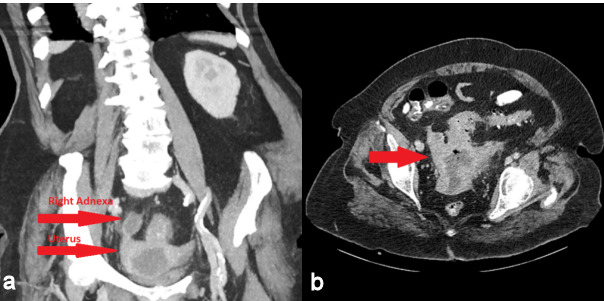
Computer tomography (CT) a) of the abdomen in the coronal plane with intravenous contrast, b) of the lower pelvis in the axial plane with intravenous contrast; air bubble in the uterine cavity and right adnexa

**Figure 3:**
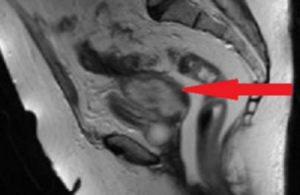
Magnetic resonance imaging (MRI) of the lower pelvis in the sagittal plane with intravenous contrast

A 72-year-old woman was admitted to our emergency department with intermittent abdominal pain, diarrhea and fever up to 38.5°C for the last three days. The patient had a history of hypertension, dyslipidemia and alcoholism. She had one vaginal delivery in the past while her gynecological history was unremarkable. Her blood pressure was 100/70 mmHg, her pulse rate was 110 beats/min and her body temperature 38°C. The physical examination revealed mild sensitivity in palpation of the lower abdomen. Laboratory studies on her admission showed a white blood cell count of 25,000/µL (normal range 4–11x10^3^/µl) with 79.3% neutrophilia (normal range 40–75%), crp 56 mg/dl (normal value <0.5) and creatinine 5.45 mg/dl (normal range 0.6–1.1 mg/dl) due to sepsis. Blood and urine culture was collected. Chest X-ray was normal. Ceftriaxone 2 gr twice daily and metronidazole 500 mg three times daily were administered promptly. The patient was observed in a high dependency unit for the first 3 days of hospitalization, but she was not intubated. The third day of her hospitalization, increased noisome vaginal discharge was observed and a gynecological assessment was requested. The optical examination of the cervix showed a purulent outflow through the cervical canal, and a pus culture was done. The pelvic exam revealed severe sensitivity in the lower pelvis. The transvaginal ultrasound examination showed an enlarged, integral uterus without any signs of perforation, hypoechoic fluid accumulation in the uterine cavity, unclear borders between endometrium – myometrium (Figure 1a (Fig. 1)), a mixed echogenic mass with a long axis of 38 mm in anatomical position of the right adnexa (Figure 1b (Fig. 1)) and no free fluid in the pouch of Douglas. The ultrasound findings were confirmed by a computed tomography (CT) with intravenous contrast, which demonstrated the presence of an air bubble in the uterine cavity, which was full of fluid, and a right tubo-ovarian abscess as well (Figure 2 (Fig. 2)). Cancer biomarkers for gynecological malignancy were negative. No bacteria grew from blood and urine cultures, whereas the vaginal pus culture grew Enterococcus faecia. The anti-bacterial medication was changed according to the antibiogram to tigecycline 50 mg iv twice daily. After three days of being fever free and improvement of the laboratory results (decrease of WBC to 11,000/mm^3^, crp to 4.83 mg/dl and creatinine to 0.71 mg/dl), the patient underwent ultrasound-guided dilatation and curettage of the cervical canal and the endometrium. The material that was collected was sent for histologic examination, which excluded malignancy. On the 10^th^ day of hospitalization, the patient underwent magnetic resonance imaging (MRI) of the lower abdomen with intravenous contrast for the purpose of evaluation of pelvic inflammation, and demonstrated concrete improvement (Figure 3 (Fig. 3)). MRI was selected as a method of follow-up scanning because it offers excellent soft tissue contrast. Since our hospital does unfortunately not provide MRI scanning, MRI occurred at a private medical center. In addition, the patient was not re-examined sonographically because there was no specialist sonographer available. After fifteen days of hospitalization, the patient was discharged in an afebrile and hemodynamically stable condition, with the recommendation of per os antibacterial medication (clindamycin 300 mg three times daily for 14 days) and a second check in two weeks. The patient did not attend her scheduled appointment.

## Discussion

Idiopathic pyometra and tubo-ovarian abscess is an infrequent event in postmenopausal women. The lack of risk factors and atypical symptoms such as abdominal pain and fever [10] could lead emergency department (ED) physicians to misdiagnosis. Delayed diagnosis may cause severe complications such as rupture of abscess, pyometra perforation and peritonitis, which require emergency surgery [3]. The differential diagnosis includes all the benign and malignant conditions that could block the cervical canal [5]. Ultrasound examination could give useful information which could be confirmed by a CT or an MRI [5], while endometrial and cervical sampling is necessary to exclude malignancy [10]. The management could be operative or conservative and depends on the etiology, the patient’s performance status, and the presence or absence of life-threatening complications [6].

Our patient has no remarkable gynecological history, no specific symptoms and no significant comorbidities which could lead to immune suppression. As far as alcoholism is concerned, human studies have largely been consistent with the well-established findings in experimental models that chronic alcohol use both predisposes to and worsens outcomes of sepsis [11]. There is one study in adolescents that suggests alcohol use as a risk factor for PID [12].

Tubovarian abscesses (TOAs) in women of reproductive age usually constitute a severe complication of acute salpingitis more often caused by N. gonorhoea and C. trachomatis. These women present with pelvic pain, fever and vaginal discharge. On the other hand, in postmenopausal women this clinical picture is usually absent. The clinical characteristics in this age group are frequently unclear and nonspecific. Furthermore, the underlying reasons present significant differences. In postmenopausal women, concomitant pelvic pathology could be found, including appendicitis, peri-appendicular abscess, acute diverticulitis, localized perforation of a diseased hollow viscus and the like in conjunction with benign or malignant conditions of the female genital system [13].

Gynecological assessment was only performed when vaginal discharge had been reported. The patient was treated conservatively since pyometra perforation or abscess rupture were excluded by imaging, and the dilatation of the cervix and drainage of the uterine contents combined with intravenous anti-bacterial administration definitely led to lysis of sepsis and the patient’s complete recovery. The dilatation of the cervical canal is the key to draining the purulent collection and preventing the abscess from rupturing into the peritoneal cavity. In combination with the intravenous administration of antibiotics, the inflammation was reduced and life-threatening complications were avoided. This fact is reflected in both the clinical picture and the laboratory exams. The repetitive imaging with MRI of the lower abdomen revealed articulate attenuation of pelvic disease. The patient was discharged on hospital day fifteen with follow-up.

## Notes

### Competing interests

The authors declare that they have no competing interests.
